# Quaternary Zinc Alloys with Magnesium, Calcium and Strontium after Hydrostatic Extrusion—Microstructure and Its Impact on Mechanical and Corrosion Properties

**DOI:** 10.3390/ma17143496

**Published:** 2024-07-15

**Authors:** Magdalena Bieda, Weronika Gozdur, Magdalena Gieleciak, Anna Jarzębska, Łukasz Maj, Łukasz Rogal, Jacek Skiba

**Affiliations:** 1 Institute of Metallurgy and Materials Science, Polish Academy of Sciences, Reymonta 25, 30-059 Krakow, Poland; w.gozdur@imim.pl (W.G.); m.gieleciak@imim.pl (M.G.); a.jarzebska@imim.pl (A.J.); l.maj@imim.pl (Ł.M.);; 2 Institute of High Pressure Physics, Polish Academy of Sciences, Sokolowska 29, 01-142 Warsaw, Poland; skiba@unipress.waw.pl

**Keywords:** biomaterials, biodegradable metals, SEM/EBSD, corrosion rate, low-alloyed zinc, plastic deformation

## Abstract

The development of bioabsorbable implants from Zn alloys is one of the main interests in the new generation of biomaterials. The main drawbacks of Zn-based materials are their insufficient mechanical properties. In the presented studies, a quaternary alloy composed of zinc with magnesium (0.2–1 wt. %), calcium (0.1–0.5 wt. %) and strontium (0.05–0.5 wt. %) was prepared by gravity casting followed by hot extrusion and then by hydrostatic extrusion. Microstructural characterization using scanning electron microscopy (SEM) and X-ray diffraction (XRD) phase analysis was performed. The mechanical properties were examined, using static tensile tests. Corrosion properties were analyzed using immersion tests. Samples were immersed in Hanks’ solution (temperature = 37 °C, pH = 7.4) for 14 days. All alloys were subjected after corrosion to SEM observations on the surface and cross-section. The corrosion rate was also calculated. The microstructure of the investigated quaternary alloy consists of the α-Zn grains and intermetallic phases Mg2Zn11, CaZn13 and SrZn13 with different grain sizes and distribution, which impacted both mechanical and corrosion properties. Thanks to the alloying by the addition of Mg, Ca, and Sr and plastic deformation using hydrostatic extrusion, outstanding mechanical properties were obtained along with improvement in uniformity of corrosion rate.

## 1. Introduction

Bioabsorbable materials constitute a new group of biomaterials that have been rapidly developed in recent years as an alternative to the permanent implants produced from titanium, stainless steel, etc. Their main goal is to fulfill their mission in the human body for a limited period of time and then degrade, producing non-toxic compounds [1]. The degradation rate is an essential factor for orthopedic implants and cardiovascular stents. Furthermore, in the case of bone implants, it eliminates additional surgery for implant removal [2]. Bioabsorbable implants for fracture repair should gradually and uniformly degrade (loss of mechanical integrity) while the bone tissue is repairing and remodeling.

The groups of metals considered for those purposes are Mg, Fe and Zn and their alloys. For bone implant applications, especially Mg alloys, were widely considered. However, not many bioabsorbable bone implants such as the MAGNEZIX^®^ interference screw are commercially available [3]. The main issue with Mg and its alloys is that they degrade too quickly and lose mechanical integrity. The interest in zinc-based alloys has gradually increased since it has been discovered that pure zinc possesses the optimal corrosion rate for bioabsorbable cardiovascular stents [1]. Design constraints for biodegradable bone fixation devices are similar [2,4]. The main drawbacks of Zn-based materials are insufficient mechanical properties [1,4,5]. The leading issues that need to be considered are static strength and ductility, cyclic fatigue, corrosion fatigue, creep and stability of these properties [5]. Extensive research, carried out in the past, proved that alloying tends to enhance the as-cast zinc’s mechanical properties significantly. Further improvement is possible only with the utilization of plastic deformation methods. Zinc, as a representative of Hexagonal Close Packed (HCP) metals, which also includes titanium and magnesium, with a limited number of slip systems and twinning possibilities, belongs to a group of hard-to-deform materials. Moreover, the low melting point of zinc implies low recrystallization temperature (close to that of room temperature) is a factor hugely influencing the cold working deformation. This means that pure zinc recovery and recrystallization processes (DRX) could be observed during deformation at room temperature. It was proved that alloying could increase the recrystallization temperature. Furthermore, the presence of second phases could also limit the DRX process, serving as an obstacle for grain boundary migration. It was additionally observed for magnesium alloys in which the influence of particle size and shape on the activation of twinning and slip systems was determined [6].

In order to improve the mechanical properties, both alloying and hot deformation methods, e.g., hot extrusion and hot rolling, were applied. Nutrition alloying elements are preferred in order to preserve the biocompatibility of the processed material. Up till now, zinc binary and ternary alloys with the addition of elements such as Mg, Cu, Ca, Sr, Mn, Ag, etc., have been comprehensively examined [3,4,5,7,8,9,10,11,12,13,14,15,16,17,18]. Among the abovementioned nutrition elements, magnesium is one of the most intensively studied zinc alloying elements. Its solubility in zinc is less than 0.1 wt.%, and in the as-cast state, it forms a eutectic mixture composed of an α-Zn and Mg_2_Zn_11_ intermetallic phase. The presence of the intermetallic phase, Mg_2_Zn_11_, formed in Zn-Mg alloys, impacts the DRX. However, this phase’s high content is not beneficial for the ductility and corrosion properties of the final material. Our previous investigation proved the profits of the synergistic effect of alloying by magnesium and plastic deformation through hydrostatic extrusion (HE) on mechanical properties and grain refinement [19,20,21,22,23].

**Figure 1:**
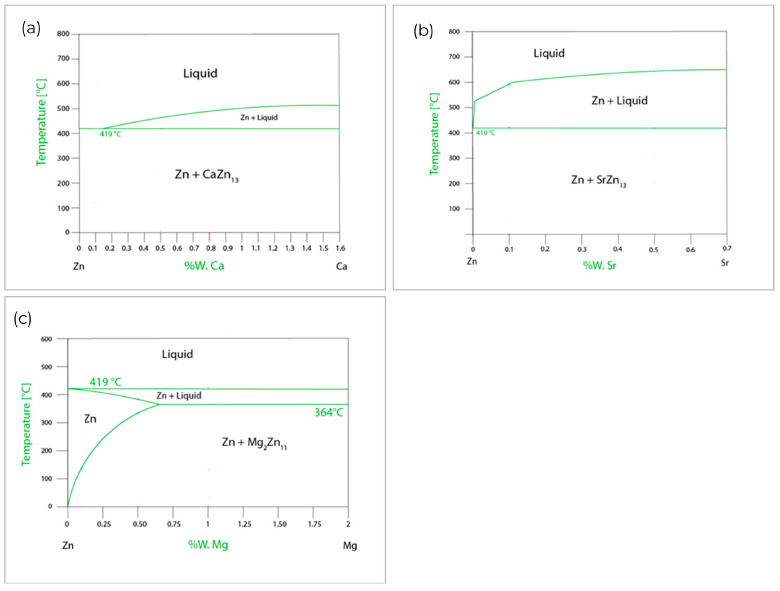
Phase equilibrium diagrams of binary systems: (**a**) Ca-Zn, (**b**) Sr-Zn and (**c**) Mg-Zn based on [26].

Several Zn-based binary alloys for biomedical applications were investigated. The most significant results were obtained for Zn alloy with the addition of Mg, Li and Ag [4,5]. Research dedicated to applying different amounts of Mg and comprehensive methods of their plastic deformation process are presented [4,5], and the achieved results are close to demand but not satisfying enough. This is why the exploration of new possibilities for alloying and deformation procedures is still needed. Several ternary systems were also studied. Among them, the addition of a small amount of Ca and Sr was considered. In striking contrast to Mg or Cu, there is no solubility of Ca and Sr in zinc, so zinc-based intermetallic phases CaZn_13_ and SrZn_13_ will form, respectively. In Figure 1. parts of binary phase diagrams are presented for the low addition of Mg, Ca and Sr to zinc. In publications [9,10], results of investigations for binary and ternary alloys with nutrient alloying elements Mg, Ca and Sr are discussed. The mechanical properties of the presented alloys are lower than required. Nevertheless, broad mechanical, corrosion and biological studies are performed for as-cast, hot rolled and hot extruded alloys, which clearly suggests good corrosion and biological performance for binary and ternary alloys with Ca and Sr addition. The addition of Ca, Sr and Mg can improve the cytocompatibility, osteogenesis, and osseointegration [14]. Advanced microstructural characterization of ZnMg0.8Ca0.2 alloys after hot extrusion is presented in [7]. The obtained mechanical properties, depending on the extrusion parameters, approach the required values, but they do not exceed them. In [8], it was noticed that although CaZn_13_ and SrZn_13_ intermetallic phases have the same crystallographic structure, their distribution, morphology, and size differ after casting, which partly explains the differences in the values of mechanical properties [9,10]. The latest publication concerned Zn alloys for orthopedic implants focuses on ZnCuTi, ZnSr or ZnMnMg systems. However, mainly biological and corrosion performance is investigated without improving those alloys’ mechanical properties using plastic deformation methods [3,16,17,18]. The need for development of the material satisfying corrosion and mechanical requirements for bone screws is evident. 

Previous investigations have shown that subjecting pure zinc and zinc alloyed with magnesium to unconventional methods of plastic deformation such as Kobo [24] and especially HE, improved their mechanical properties to a level unattainable for other methods [18,19,20,21,22,23]. For the conventional methods, alloying by magnesium improved mechanical properties but decreased ductility (although, in the case of HE with a higher addition of Mg, the ductility was improved [19]). However, HE caused the formation of a band-like microstructure, where primary α-Zn grains are alternately arranged with bands of a eutectic mixture, which due to the different potential of pure metal and intermetallic phase may result in differences in degradation rate. The corrosion pitting could be harmful to the mechanical integrity of the implant for the time needed for remodeling the damaged tissue [25]. 

The present study examined to what extent the processes of multiple alloying with alloying additives such as Ca, Mg, Sr and plastic deformation using hydrostatic extrusion affect the mechanical properties and corrosion rates of material from the group of biodegradable alloys with potential application for bone implants. For the first time, the quaternary alloys with these components after hydrostatic extrusion are considered.

## 2. Materials and Methods

### 2.1. Material Processing

For the experiments described in this paper, samples made of quaternary ZnMgCaSr alloys with three different chemical compositions summarized in Table 1 were used. The alloys were labeled: ZnMgCaSr_1, ZnMgCaSr_2 and ZnMgCaSr_3. 

**Figure 2:**
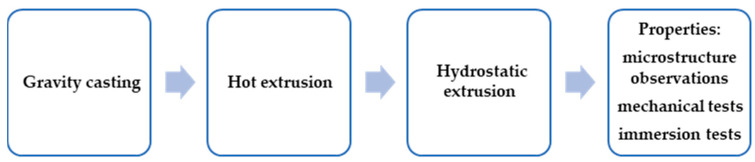
Diagram of the manufacturing and testing process of the alloys.

For individual alloys, intermetallic phases are predicted to occur following what is seen in individual zinc phase equilibrium systems (Figure 1) with alloying additives. Materials with the compositions specified above were formed according to the manufacturing process, which is shown in a simplified manner in the logic diagram (Figure 2).

Gravity casting of the alloys was carried out using steel molds in molten components in a Naberthem N20/14 resistance furnace under an argon atmosphere. Pure commercially available alloy components were used: Zinc (purity 99.99%), Magnesium (purity 99.95%), Strontium (purity 99%) and Calcium (purity 99%). In each case, purity is expressed in mass percentage. The composition of the alloys was confirmed using optical emission spectrometry (ICP-OES) presented in Table 1.

In an attempt to determine what effect the applied plastic processing has, the various compositions described above were subjected to hot extrusion at 250 °C. The final stage of material preparation was a hydrostatic extrusion carried out at room temperature in two passes with cumulative strain *ε_cum_*  =  1.4. Hydrostatic extrusion is a method of extrusion of the billet under high pressure in the liquid environment conducted in HE-presses able to operate at pressures up to 2.5 GPa designed and constructed at the Institute of High Pressure Physics Polish Academy of Science. 

### 2.2. Mechanical Properties—Static Tensile Test 

A static tensile test was used to determine the mechanical properties. Three specimens each representing the alloys analyzed in the work were tested on a Zwick/Roell Z250 kN machine. On that basis, tensile curves were determined showing the course of changes occurring inside the material during uniaxial deformation of the standardized cylindrical samples with a 3 mm in diameter and a gauge length of 15 mm.

For the rupture process itself, an initial force of 20 N was pledged. The specimens were deformed at a speed of 0.008 s^−1^. Tensile strength, yield strength and elongation to failure were determined. 

### 2.3. Corrosion Properties—Static Corrosion Test

A static immersion test was conducted to determine the corrosion properties of the four-component alloys. For the test, nine samples in the form of cylinders with a total surface area of 0.7056 cm^2^ were cut from each analyzed material. In each case, six of them were used for weight loss testing, while the remaining three for SEM microscopic observations. Before the test, the prepared material was cleaned using an ultrasonic cleaner and weighed. The prepared samples were placed individually in sterile containers and then flooded with corrosion medium and kept in an incubator at 37 °C, Hanks’ salt solution (HBSS) was used as the corrosion medium. The test lasted 14 days, to keep the pH of the corrosion solution at a certain level (±7) it was replaced every 48 h. At each replacement, the pH of the freshly added solution and the solution in the containers was measured. In addition, pH changes were observed also for the empty container. At the end of the experiment, the samples were elevated from the corrosion medium and then weighed. In addition, the samples intended for the determination of corrosion rates were subjected to a procedure for cleaning the corrosion products with chromic (VI) acid for 20 min at room temperature. Finally, they were dried and weighed using an analytical balance with a resolution of 0.1 mg. The data thus obtained were used to determine the corrosion rate according to the Equation (1):

(1) $$V_{k}=\frac{8.74\cdot 10^{4}\cdot W}{x\cdot t\cdot \rho }$$

where *V_k_* stands for corrosion rate, *W* is a mass loss, *x* is the surface of the sample, *t* represents time of test and *ρ* is the density of material. 

### 2.4. Microstructural Observations

X-ray diffraction (XRD) phase analysis was performed by using Bruker D8 Discover equipped with a Co anode and ICDD PDF-4+ database. Scanning electron microscopy observation using Quanta 3D FEG (Thermo Fisher, Eindhoven, The Netherlands) scanning electron microscope with the Energy Dispersive X-ray Spectroscopy (EDS) and Electron Backscatter Diffraction (EBSD) (EDAX Amtec, Pleasanton, CA, USA) detector were extensively used for the characterization of the samples after deformation and immersion test. EBSD maps were acquired with the dimensions of 200 × 200 µm and 120 × 120 µm with step sizes 200 and 100 nm, respectively, for the material after hot and hydrostatic extrusion. SEM/EBSD data were analyzed using TSL OIM ver. 7.2 analysis software (EDAX Amtec, USA) only for grains indexed as belonging to a HCP Zn phase. Pixels corresponding to the intermetallic phases were excluded from the calculations. The EDS elemental mapping together with the Image quality parameter were used for phase differentiation. The intermetallic phases were marked black in color in the orientation maps. The grain was defined as a set of at least five measurement points surrounded by a continuous grain boundary segment with a misorientation of at least 15°. Average grain size was represented as a measurement average grain diameter of a circle with an equivalent surface area. The arithmetic average (“number”) and weighted average (“area”) grain sizes, where the grain size is the weight (number and area) were calculated for each map. Samples were cut from a cross-section of a bar parallel and longitudinal to the extrusion direction, called respectively, transverse (TS) and longitudinal (LS) cross sections. The polishing process was carried out on automatic polishers using diamond suspensions with a particle size of 1 μm and ¼ μm. The final stage of preparation was electrochemical polishing at 25 °C. The samples were treated with Struers’ C1 electrolyte for 12 s. Cross-sections from the samples after immersion tests were embedded in the resin and polished. A protective layer of carbon was used for SEM observations. 

## 3. Results and Discussion

### 3.1. Mechanical Properties

**Figure 3:**
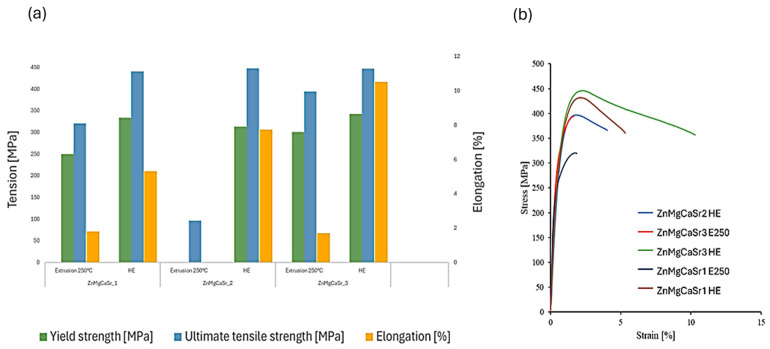
Mechanical properties (**a**) (yield strength, ultimate tensile strength and elongation to failure) with exemplary strain-stress curves (**b**) obtained for ZnMgCaSr_1, ZnMgCaSr_2 and ZnMgCaSr_3 after hot extrusion (E250) and hydrostatic extrusion (HE).

Based on the static tensile test mechanical properties such as tensile strength, yield strength, and elongation to failure were determined. In the case of tensile strength for each analyzed material, it is noticeable that it increases with successive stages of plastic processing. The second parameter determined was yield strength. Here, as in the case of tensile strength, the value increases with successive treatments. The highest result was obtained for the ZnMgCaSr_3 alloy subjected to hydrostatic extrusion. Due to immediate failure during the test, it was not possible to determine the yield strength and relative elongation for the ZnMgCaSr_2 alloy after hot extrusion. The material (ZnMgCaSr) with the highest content of alloying elements (1.5%) after hot extrusion at 250 °C is brittle, however further deformation by hydrostatic extrusion improves all mechanical properties of the investigated alloys which are often observed for the hard-to-deformed alloys subjected to the hydrostatic extrusion [27]. From the results obtained, a trend can be observed, indicating that elongation to failure always increases with successive stages of plastic deformation. For ZnMgCaSr_3 alloy subjected to hydrostatic extrusion, a significant increase in ductility is observed which is in agreement with previously reported e.g., [19]. Analyzing the alloys in terms of chemical composition, an increase in Mg content and a decrease in Ca content contributed to an increase in ductility of the analyzed alloys. The stress-strain curves revealed typical for Zn alloys strain-softening phenomenon [28]. The summary of obtained results is in bar chart form shown in Figure 3. 

The received mechanical properties summarized in Table 2 are comparable with ultimate tensile strength (UTS) and yield strength (YS) for all the investigated alloys meeting the requirements for bioabsorbable implants for orthopedic applications especially bone screws except for the alloy with the lowest content of magnesium (ZnMgCaSr_1) with the lowest elongation to failure [28].

### 3.2. Microstructural Characterization

**Figure 4:**
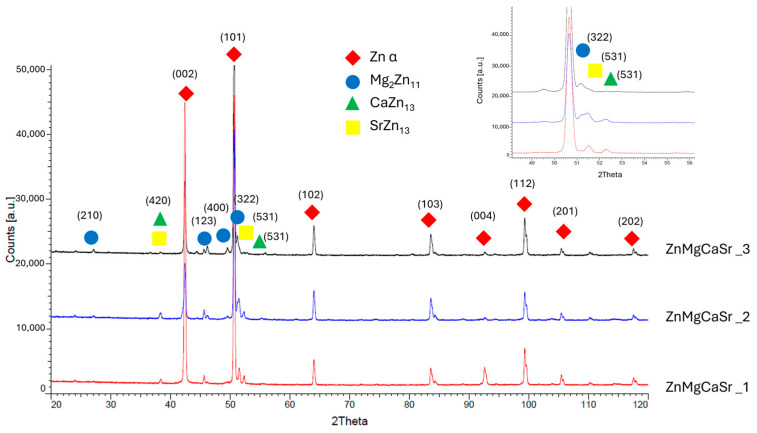
XRD diffractogram of Zn alloys with different compositions ZnMgCaSr_1, ZnMgCaSr_2 and ZnMgCaSr_3, with marked peaks from intermetallic phases and pure zinc and insert with higher magnifications.

**Figure 5:**
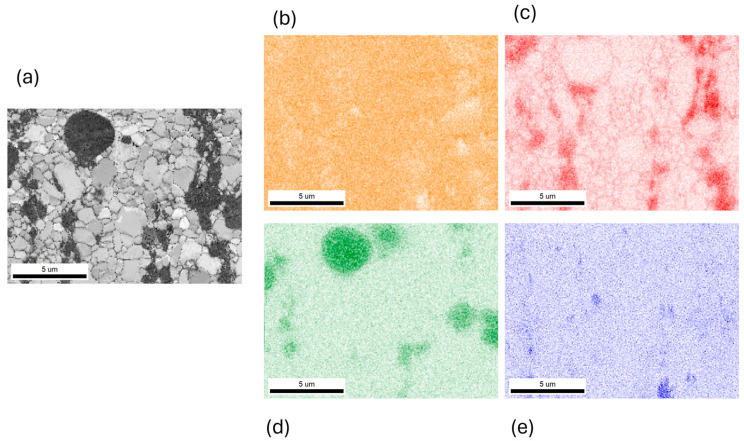
Exemplary high magnifications EDS/EBSD analysis of the investigated Zn alloys with (**a**) IQ map and elements distribution maps of Zn (**b**), Mg (**c**), Ca (**d**) and Sr (**e**).

The specified mechanical properties of materials are directly related to their microstructure and, consequently, to the grain size of the materials and the distribution and refinement of second-phase particles. XRD phase analysis (Figure 4) confirmed the presence of all four phases in the investigated materials including the α-Zn grains and intermetallic phases Mg_2_Zn_11_, CaZn_13_ and SrZn_13_. Furthermore, based on microstructural observations using the EBSD (electron backscatter diffraction) method and EDS, maps were obtained showing the shape and distribution of all phases. It is visible (Figure 5) that CaZn_13_ formed the largest particles isolated with rectangular or oval shapes, while the Mg_2_Zn_11_ and SrZn_13_ formed refinement grains mainly round-shaped and often in the near vicinity to each other, e.g., [6]. 

The first stage of deformation of the materials was hot extrusion at 250 °C. The effect of this treatment was the formation of a coarse-grained microstructure in each analyzed alloy. For all analyzed compositions, the grains after extrusion were slightly elongated in the extrusion direction. The largest grain size was presented for the ZnMgCaSr_3 alloy on both longitudinal and transverse cross-sections, while the smallest grain size was presented for the ZnMgCaSr_2 alloy.

**Figure 6:**
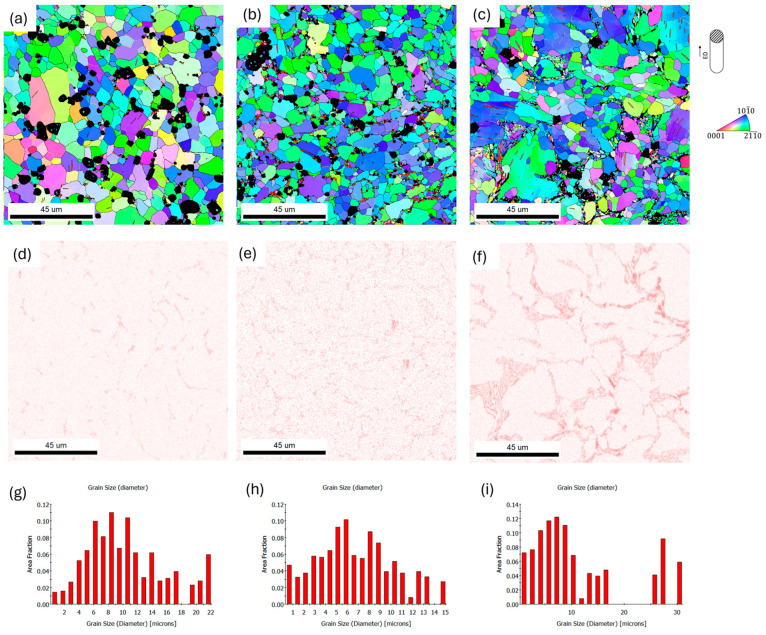
SEM/EBSD IPF color-coding orientations maps for hot extruded at 250 °C low-alloyed zinc (**a**) ZnMgCaSr_1, (**b**) ZnMgCaSr_2 and (**c**) ZnMgCaSr_3, EDS elemental maps of magnesium content for (**d**) ZnMgCaSr_1, (**e**) ZnMgCaSr_2, (**f**) ZnMgCaSr_3; grain size distribution for (**g**) ZnMgCaSr_1, (**h**) ZnMgCaSr_2 and (**i**) ZnMgCaSr_3, transverse cross sections. The black areas on the orientation maps correspond to the intermetallic phases, excluded from the calculations, the inverse pole figure (IPF) is shown with respect to the extruded direction.

**Figure 7:**
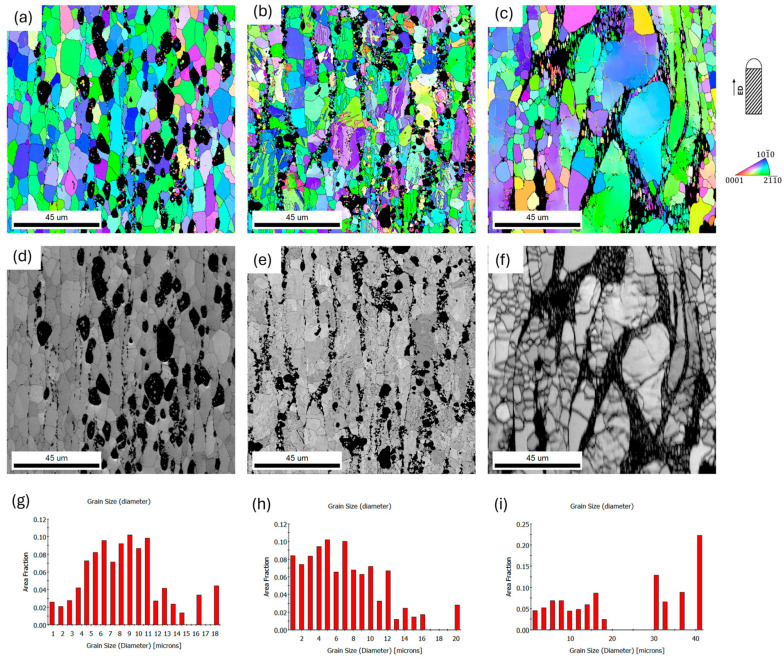
SEM/EBSD IPF color-coding orientations maps for hot extruded at 250 °C low-alloyed zinc (**a**) ZnMgCaSr_1, (**b**) ZnMgCaSr_2 and (**c**) ZnMgCaSr_3, IQ maps for (**d**) ZnMgCaSr_1, (**e**) ZnMgCaSr_2, (**f**) ZnMgCaSr_3; grain size distribution for (**g**) ZnMgCaSr_1, (**h**) ZnMgCaSr_2 and (**i**) ZnMgCaSr_3, longitudinal cross section. The black areas on the orientation maps correspond to the intermetallic phases, excluded from the calculations, the inverse pole figure (IPF) is shown with respect to the extruded direction.

For ZnMgCaSr_3 alloy the bimodal distribution of the grain size was observed in Figure 6 and Figure 7. The largest grains were over 30 µm while the smaller ones were less than 20 µm. For the other two alloys with less magnesium content, a more uniform distribution of the grain size was observed for both cross-sections of the extruded rods (Figure 6 and Figure 7). It is related to the distribution of the magnesium intermetallic phase, which is shown in EDS elemental maps in Figure 6d–f. It is also visible that refinement of other intermetallic phases with Sr and Ca when the amount of magnesium is larger. For ZnMgCaSr_1, the CaZn_13_ and SrZn_13_ phases are larger and especially, CaZn_13_ could reach more then 20 µm (Figure 7d).

**Figure 8:**
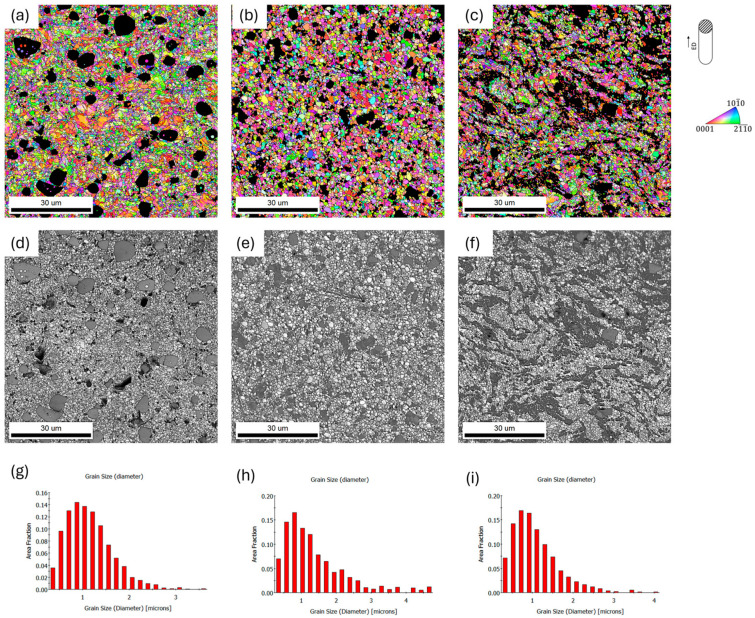
SEM/EBSD IPF color-coding orientations maps after two passes of hydrostatic extrusion of low-alloyed zinc (**a**) ZnMgCaSr_1, (**b**) ZnMgCaSr_2 and (**c**) ZnMgCaSr_3. IQ maps for (**d**) ZnMgCaSr_1, (**e**) ZnMgCaSr_2, (**f**) ZnMgCaSr_3; grain size distribution for (**g**) ZnMgCaSr_1, (**h**) ZnMgCaSr_2 and (**i**) ZnMgCaSr_3, transverse cross section. The black areas on the orientation maps correspond to the intermetallic phases, excluded from the calculations, the inverse pole figure (IPF) is shown with respect to the extruded direction.

**Figure 9:**
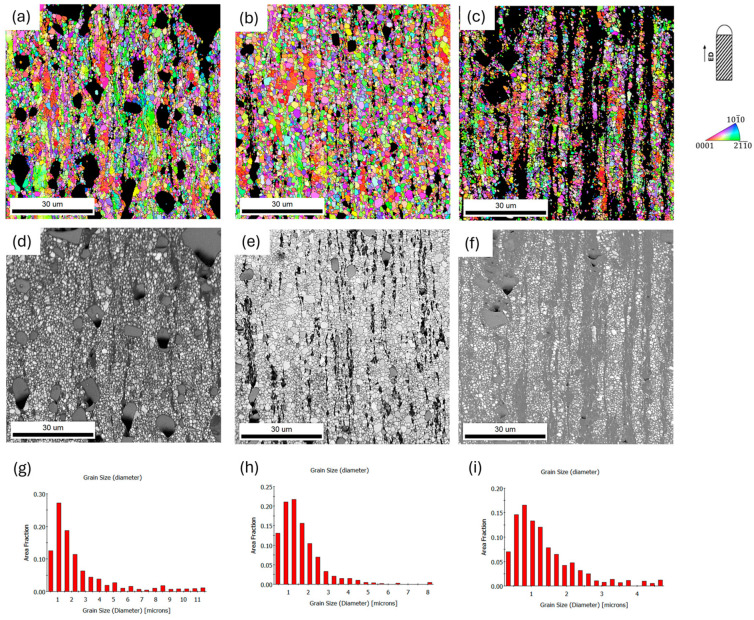
SEM/EBSD IPF color-coding orientations maps after two passes of hydrostatic extrusion of low-alloyed zinc (**a**) ZnMgCaSr_1, (**b**) ZnMgCaSr_2 and (**c**) ZnMgCaSr_3, IQ maps for (**d**) ZnMgCaSr_1, (**e**) ZnMgCaSr_2, (**f**) ZnMgCaSr_3; grain size distribution for (**g**) ZnMgCaSr_1, (**h**) ZnMgCaSr_2 and (**i**) ZnMgCaSr_3, longitudinal cross sections. The black areas on the orientation maps correspond to the intermetallic phases, excluded from the calculations, the inverse pole figure (IPF) is shown with respect to the extruded direction.

Further refinement of the grain size was observed after two passes of hydrostatic extrusion. The average grain size in this case was in the range of 0.63–2.1 μm, and depends on the parameter we chose (Table 3). The finest microstructure was characterized by the ZnMgCaSr_2 alloy and ZnMgCaSr_3 alloy. What is visible is that grains have similar sizes on both cross sections, for those behaviors, the DRX process during hydrostatic extrusion is responsible [19,20]. Moreover, the difference in size and distribution of intermetallic phases Mg_2_Zn_11_, CaZn_13_ and SrZn_13_ was recognized. The Mg_2_Zn_11_ phase formed a characteristic eutectic mixture and elongated bands in the extrusion direction. They are much wider for the ZnMgCaSr_3 alloy. This was also observed in [19,20,21]. However, for the other two intermetallic phases, the most refinement and uniform distribution of those phases was observed for the ZnMgCaSr_2 alloy. This could be crucial for the homogeneous mechanical and corrosion behavior of these alloys for planned applications. Figure 8 and Figure 9 show the EBSD maps for the analyzed alloys. From the above results, it is noticeable that there is a significant effect of plastic processing on the refinement of the structure for all analyzed alloys. With each successive processing step, an increasingly fine-grained structure was present in all investigated materials.

### 3.3. Corrosion Rate

**Figure 10:**
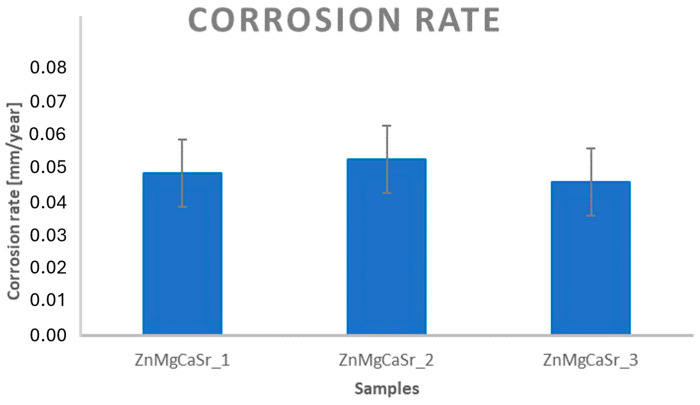
Diagram of corrosion rates calculated for ZnMgCaSr_1, ZnMgCaSr_2 and ZnMgCaSr based on immersion tests.

**Figure 11:**
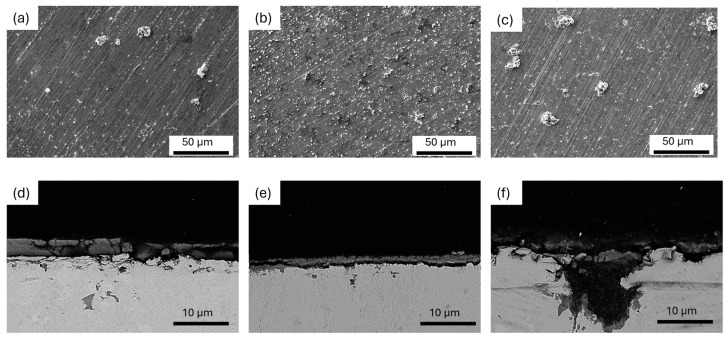
SEM images of the (**a**–**c**) top surface, (**d**–**f**) and cross-section of the samples after immersion tests ZnMgCaSr_1 (**a**,**d**), ZnMgCaSr_2 (**b**,**e**) and ZnMgCaSr_3 (**c**,**f**) alloys.

Based on the immersion test carried out for 14 days, it was possible to determine the corrosion rate for the analyzed alloys. Figure 10 shows the V_k_ [mm/year] for the various alloys analyzed. It is visible that the value of the corrosion rate is similar for all investigated materials but also similar for other Zn alloys investigated with the same immersion test parameters presented e.g., in [29]. The sample weights (based on six measurements) with the same composition differ from each others with the same amount as between different compositions. However, interesting results were obtained after observation of the samples using scanning electron microscopy. The corrosion products are expected to be present on the surface of the samples. Figure 11a–c shows images of the surface after the immersion test. As expected, clusters of corrosion products are visible on the surface of each analyzed sample. They are spontaneous without a schematic pattern of occurrence. Analyzing the number of clusters, it is noticeable that a significant predominance of them occurs for the ZnMgCaSr_2 alloy after hydrostatic extrusion. The clusters are smaller but occur in great numbers. In contrast, the other two alloys show the occurrence of larger and less frequent corrosion clusters. The observation of a cross-section of the samples after corrosion indicates a large difference in the corrosion layers. The large amount of magnesium phases for the ZnMgCaSr_3 alloy is manifested with intensive corrosion pits (Figure 11f), which was also observed, e.g., in [6]. For the other two samples, corrosion pits even if present are very shallow, which corresponds to greater dispersity of the intermetallic phases.

The obtained results reveal the correspondence between the microstructure and the properties of the materials and show how critical is microstructural characterization for a deeper understanding of the behavior of the materials especially for such important applications. From the results of mechanical properties and corrosion rate calculations, there are no huge differences between the investigated quaternary alloys with different amounts of elements such as Mg, Ca and Sr. However, microstructural characterization revealed the difference in the phase’s size and distribution which will affect the uniformity of mechanical integrity of the implant produced from those alloys.

## 4. Conclusions

The development of bioabsorbable implants from Zn alloys is one of the main interests in the new generation of bioabsorbable alloys. From the point of excellent corrosion properties and good biocompatibility in the presented state of the art, there is only one problem that makes these alloys far from the market and final products, and this is their mechanical properties. Thanks to the synergy of proper alloying (by the addition of Mg, Ca, and Sr) and using an accurate deformation method (hydrostatic extrusion preceded by hot extrusion), these properties can be obtained. Implants from such material will be a suitable replacement for permanent implants from stainless steel (SS), cobalt alloys, titanium (Ti) and Ti alloys available on the market now. It will be competitive with other bioresorbable materials such as magnesium alloys and polymers.

Based on the results of the presented work, the following conclusion can be stated:

- Hydrostatic extrusion of the quaternary zinc alloys leads to a large improvement in their mechanical properties. The obtained UTS and YS are on the same high level, regardless of the composition of the alloys when elongation rose with the amount of magnesium added.
- Increasing the content of magnesium as an alloying additive may have contributed to greater material refinement, but also changed the distribution of the intermetallic phases, which also became more refined, especially for Mg_2_Zn_11_ and SrZn_13_.
- The corrosion rate had comparable values, but observations of the surface and cross sections of the samples after corrosion tests indicated a relationship between magnesium content and the occurrence of corrosion pitting in the material caused by the intermetallic phase.
- The alloys with the highest amount of elements added possess the best balance between uniform distribution of the intermetallic phase and grain refinement, as seen by the most uniform corrosion with the smallest observed corrosion pits and improvement in mechanical properties satisfying the requirements for bone screws.

## Figures and Tables

**Table 1:** Alloy composition based on ICP-OES in wt. %; zinc in balance.

	Mg	Ca	Sr
ZnMgCaSr_1	0.225 ± 0.020	0.567 ± 0.046	0.0558 ± 0.0045
ZnMgCaSr_2	0.522 ± 0.045	0.495 ± 0.042	0.536 ± 0.046
ZnMgCaSr_3	1.012 ± 0.090	0.122 ± 0.010	0.0103 ± 0.0009

**Table 2:** Mechanical properties of the investigated alloys.

Mechanical Properties	ZnMgCaSr_1		ZnMgCaSr_2		ZnMgCaSr_3	
	Hot Extrusion 250 °C	Hydrostatic Extrusion	Hot Extrusion 250 °C	Hydrostatic Extrusion	Hot Extrusion 250 °C	Hydrostatic Extrusion
YS [MPa]	249 ± 3	324 ± 13	-	314 ± 23	305 ± 5	343 ± 12
UTS [MPa]	320 ± 1	435 ± 5	96.3 ± 20	447 ± 1	399 ± 5	444 ± 3
Elongation [%]	1.8 ± 0.2	5.3 ± 0.2	-	7.8 ± 0.5	1.6 ± 0.1	10.4 ± 0.1

**Table 3:** Average grain size of the investigated alloys.

Average Grain Size ± Standard Deviation [µm]	ZnMgCaSr_1		ZnMgCaSr_2		ZnMgCaSr_3	
	Number	Area	Number	Area	Number	Area
TS after hot extrusion	2.08 ± 3.16	10.46 ± 5.33	1.39 ± 1.80	6.75 ± 3.52	1.76 ± 2.40	11.38 ± 9.17
LS after hot extrusion	2.35 ± 2.81	8.46 ± 3.96	1.64 ± 1.86	7.00 ± 4.46	3.17 ± 4.68	23.26 ± 14.60
TS after hydrostatic extrusion	0.76 ± 0.36	1.14 ± 0.49	0.68 ± 0.42	1.36 ± 0.90	0.63 ± 0.34	1.07 ± 0.57
LS after hydrostatic extrusion	0.94 ± 0.66	2.55 ± 0.66	0.81 ± 0.51	1.61 ± 1.06	0.68 ± 0.42	1.36 ± 0.90

## Data Availability

The original contributions presented in the study are included in the article, further inquiries can be directed to the corresponding authors.
